# Recurrence of Pleomorphic Adenoma in the Submandibular Gland: A Case Report and Literature Review

**DOI:** 10.2174/0115734056333595250226071534

**Published:** 2025-03-25

**Authors:** Zhiqiong Li, Guiying Yuan, Ye Zhang, Junbin Huang, Fan Xu, Yuchao Xiong, Xuwen Zeng

**Affiliations:** 1 Department of Radiology, Guangzhou Red Cross Hospital (Guangzhou Red Cross Hospital of Jinan University), 396 Tongfu Road Guangzhou, Guangdong Province, 510220, China; 2 Department of Respiratory, Guangzhou Red Cross Hospital (Guangzhou Red Cross Hospital of Jinan University), 396 Tongfu Road Guangzhou, Guangdong Province, 510220, China

**Keywords:** Pleomorphic adenoma, Submandibular gland, Recurrence, Magnetic resonance imaging, Computed tomography, Case reports, Submandibular gland pleomorphic adenoma, Benign tumor, Diffusion-weighted imaging

## Abstract

**Introduction:**

Recurrent pleomorphic adenoma (PA) in the submandibular gland is a rare tumor that may be misdiagnosed as an inflammatory lesion. The imaging manifestations of the submandibular gland recurrent PA are unclear, with only three case reports reporting CT and MRI imaging, respectively. Our report is the first case report that comprehensively describes the imaging manifestations of recurrent PA in the submandibular gland.

**Case Presentation:**

A 28-year-old woman had a right submandibular gland pleomorphic adenoma that recurred 5 years after resection and gradually grew larger. She had no special discomfort and was diagnosed with a recurrence of pleomorphic adenoma. The patient underwent CT and MRI examinations and tumor resection, and postoperative pathology showed tumor recurrence.

**Conclusion:**

This case report provides substantial and comprehensive CT and MRI data, which is conducive to the diagnosis of the recurrence of submandibular gland pleomorphic adenoma and the avoidance of misdiagnosis to the greatest extent possible.

## 1 INTRODUCTION

Pleomorphic adenoma (PA) is the most prevalent benign salivary gland tumor, with a predilection for middle-aged females. It originates from the major salivary glands, although it may also arise from the minor salivary glands [1]. The parotid glands are the most frequently affected (85%), followed by the minor salivary (10%) and submandibular glands (5%). The recognition of PA is conceptualized by identifying three components: epithelial, myoepithelial, and mesenchymal. Epithelium in a loose fibrous stroma of myxoid, chondroid, or myxoid type shows different patterns in the histological manifestations of PA [2]. Although benign, these epithelial tumors have a tendency to relapse and become malignant if not completely resected, which can result in increased morbidity in these patients [1]. There are few imaging reports of recurrent PA in this site, and the imaging description is not detailed. Therefore, we report a case of recurrent PA in the right submandibular gland and review the literature, focusing on its imaging features, differential diagnosis, and possible factors for recurrence.

## 2 CASE REPORT

A 28-year-old woman was admitted to the hospital with a diagnosis of a right submandibular mass that had re-swelled 5 years after the initial surgical removal of the tumor. The patient had undergone a resection of the right submandibular mass at another hospital 10 years prior. The postoperative pathological result of this case was considered to be a mixed tumor. Five years ago, the tumor recurred for no apparent reason, approximately the size of a soybean. Thereafter, the tumor gradually increased in size, and the B-ultrasound demonstrated that the right submandibular gland was occupied by solid nodules, prompting the consideration of a mixed tumor.

A specialist physical examination revealed a localized submandibular bulge on the right side, a surgical scar of approximately 2 cm on the surface, and a palpable oval mass of 3.0×2.0 cm. It was firm, non-tender, with clear margins, an acceptable range of motion, and no surface damage, ulceration, or pus. Multiple lymph nodes were palpated in areas II and III on the right side of the neck, which had clear margins and no tenderness, with a maximum size of approximately 1.0 × 1.0 cm.

**Fig. (1):**
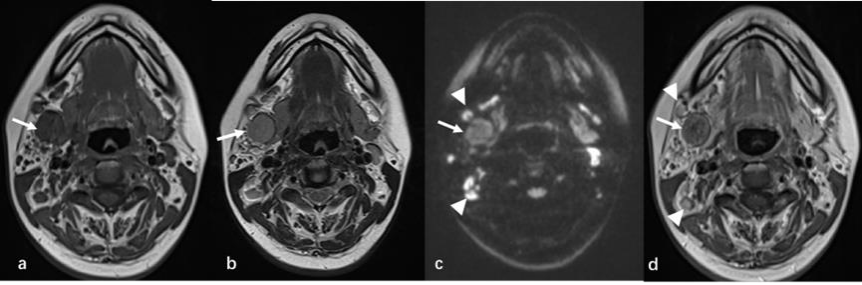
On MRI, the right submandibular gland was reduced in size, showing postoperative changes. Multiple round nodules (arrow) were observed in the posterior and inferior regions of the right submandibular gland. (**a**) The submandibular gland nodules had a moderately to slightly low signal on axial T1-weighted images and (**b**) a slightly high signal on axial T2-weighted images. (**c**) On axial DWI, the nodules had a high signal, with multiple scattered lymph nodes visible in the surrounding area (arrowhead). (**d**) On axial contrast-enhanced MRI, the nodules showed heterogeneous enhancement. The unenhanced necrotic area was observed in the center of the nodules, and capsule signs appeared around it. The peripheral lymph nodes exhibited significant enhancement (arrowhead).

The patient underwent magnetic resonance imaging (MRI) on the first day of admission. There were multiple round nodules in the posterior and inferior regions of the right submandibular gland. T1-weighted images showed an intermediate or slightly low signal and T2-weighted images exhibited a slightly high signal. The largest was approximately 1.8×2.2×2.8 cm in size. The signal was slightly heterogeneous. Diffusion-weighted imaging (DWI) demonstrated a high signal, and apparent diffusion coefficient (ADC) mapping showed a slightly high signal. The contrast-enhanced scans exhibited non-uniform enhancement. The unenhanced necrotic area was seen in the center of the nodules, and a capsule sign appeared around it. A number of enlarged lymph nodes were observed on the right side of the neck, with notable enhancement. The largest of these was approximately 1.0 cm in size (Fig. **1**). Based on the MR imaging features, a recurrence of a PA of the right submandibular gland was suspected.

**Fig. (2):**
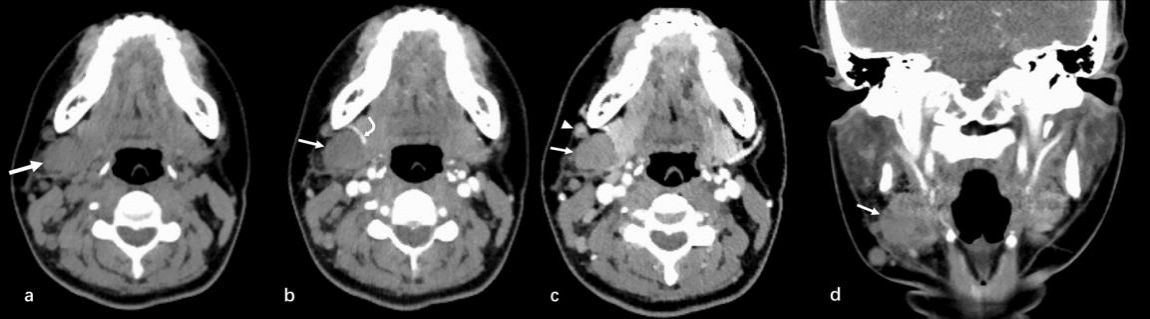
(**a**) Axial non-contrast CT showed multiple round low-density nodules (arrow) in the posterior and inferior regions of the right submandibular gland, some of which were clustered. Some of the nodules exhibited clear borders, while others displayed unclear borders with the right submandibular gland. (**b**, **c**, and **d**) On contrast-enhanced axial and coronal CT, the nodules demonstrated slight to moderate enhancement, with heterogeneous enhancement. The peripheral blood vessel was displaced anteriorly (curved arrow). The adjacent cervical fascia was slightly thickened. Multiple scattered lymph nodes were seen around the nodules and showed significant enhancement (arrowhead).

On the second day, a non-contrast computed tomography (CT) of the neck and a contrast-enhanced CT of the neck were performed. Multiple round, low-density nodules were observed in the posterior and inferior regions of the right submandibular gland, some of which were clustered. The largest was about 2.3 ×1.8 cm in size. Some of the nodules exhibited clear borders, while others displayed unclear borders with the right submandibular gland. The enhancement was of a relatively mild to moderate degree and exhibited slight unevenness. The adjacent cervical fascia was slightly thickened. Multiple scattered enlarged lymph nodes were seen on the right side of the neck, ranging from approximately 0.3 to 1.1 cm in diameter. The enhancement was not homogeneous (Fig. **2**). Based on the CT imaging characteristics, tumor recurrence was considered.

**Fig. (3):**
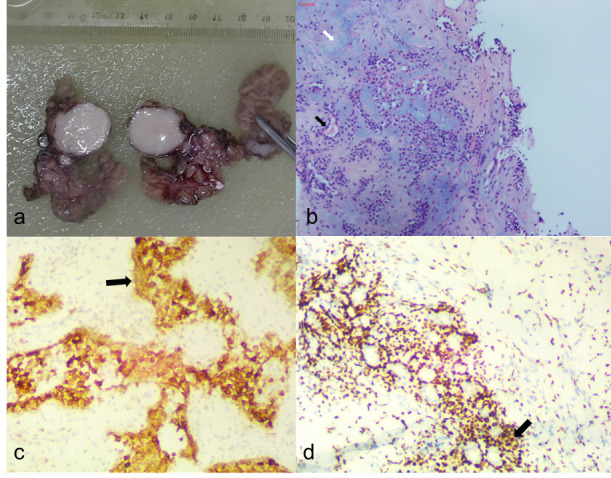
(**a**) Gross pathology specimen. The greyish-red submandibular gland tissue was submitted for examination. Upon incision, multiple greyish-white solid nodules were observed, with distinct borders and a maximum diameter of 0.5 cm to 2.5 cm. (**b**) The H&E stain at 40× magnification revealed multiple tumor structures, including ductal epithelium (black arrows) and myxoid and chondroid matrix (white arrows). (**c**) Immunohistochemical images at 100x magnification showed CK (Pan) (+) (black arrows). (**d**) Immunohistochemical images at 100x magnification showed P63 (+)(black arrows).

On day 2, a puncture biopsy of the right submandibular gland tumor was performed. The result was a PA of the right submandibular gland. The patient underwent “right submandibular gland and mass resection, cervical lymph nodes resection and arbitrary skin flap arthroplasty” in our hospital. The greyish-red submandibular gland tissue was sent for examination, and through the incision, multiple greyish-white solid nodules with tough, clear borders and maximum diameters of 0.5 cm-2.5 cm were seen (Fig. **3**).

Under the microscope, multiple nodules were seen, with nodules of different sizes and fibrous capsules. They were composed of epithelium, myoepithelial-like cells, mucus, and bone-like matrix (Fig. **3**). The cells were arranged in glandular or sheet-like shapes, and some epithelial cells were actively proliferating. Some epithelial proliferation was active with mild to moderate atypical hyperplasia. In some regions, fibrous tissue hyperplasia and hyaline degeneration involved surrounding adipose tissue and muscle fibers. In some of the lymph nodes sent for examination, residual lymphoid tissue was found around the tumor, and it was highly suspected that the tumor involved the lymph nodes. Immunohistochemical analysis revealed P63 (+), S100 (+), GFAP (-), CK (Pan) (+), BCL-2 (+), SMA (-/+), P53 (about 20%+), and Ki-67 (about 5%+). The pathological diagnosis of the right submandibular gland tumor was a PA (recurrent, multifocal distribution). This PA was accompanied by mild to moderate atypical hyperplasia and had malignant potential with the possibility of malignant transformation and metastasis.

## 3 DISCUSSION

PA is the most common benign tumor of the submandibular gland. The preferred treatment for PA of the submandibular gland is “complete” resection of the tumor and the affected submandibular gland. The good postoperative oncologic outcome of PA of the submandibular gland contrasts with the well-known great risk of recurrence of PA of the parotid gland. Recurrence of PA in the submandibular gland is rare, and Mantsopoulos [3, 4] reported a total of 147 cases of PA in the submandibular gland, none of which recurred. Due to its rarity, imaging manifestations have not been systematically reported.

To the best of our knowledge, only 15 cases of pathologically confirmed recurrent PA in the submandibular gland have been reported in the English literature [5-9], and only 3 of them mentioned the imaging manifestations of recurrent PA in the submandibular gland. Grabovac [6] and Yasumoto [9] reported only the CT manifestations of recurrent PA in the submandibular gland, and İnan [7] reported only the MRI manifestations of recurrent PA. Our report is the first case report that comprehensively describes the imaging manifestations of recurrent PA in the submandibular gland.

Most reported primary PA tumors are solitary masses [10], but recurrent PA tumors often present as multiple nodules, whether they occur in the parotid gland or submandibular gland. Phillips *et al*. [9a] reported 126 patients with recurrent parotid PA, of whom 85 (67.4%) presented with multinodular lesions. Two previously reported [6, 7] cases of recurrent PA occurring in the submandibular gland also showed multi-nodularity, and the imaging of the present case was consistent with the previous reports, showing a multinodular lesion. Recurrent PA of the parotid gland presenting as multinodular is thought to be associated with pseudocapsular rupture and inadvertent tumor spillage. Therefore, it is suggested that recurrent submandibular PA presenting as multinodular may be similarly associated with pseudocapsular rupture and inadvertent tumor spillage [11]. In addition, the possibility of recurrence in the glandular and ductal tissue surrounding the submandibular gland cannot be ruled out.

Primary PA has well-defined borders, and lesions are usually lobulated [10]. Yasumoto *et al*. [9] stated that recurrent PAs, like most primary tumors, have clear contours and smooth margins. However, in this case, most borders of the tumor were clear, and a small portion of the borders were unclear. Therefore, the pathology suggested that this case was accompanied by mild to moderate atypical hyperplasia with some malignant potential. Therefore, when the borders of recurrent PA are unclear, one should be alert to the possibility of malignancy. The recurrent PA, in this case, showed a slightly higher signal on T2-weighted images, which was not consistent with the apparent high signal on T2 shown in the images provided in the case report by İnan *et al*. [7]. This may be due to different tissue subtypes. Histopathologically, PA can be classified into three types: mucinous (or stroma-rich), cellular, and classic [3]. Among recurrent PA, the myxoid subtype has been shown to be the most prevalent, with myxoid material showing a markedly high signal on T2WI, whereas the other types, with a lower proportion of myxoid material, may show a slightly higher signal on T2WI.

An enhancement pattern was recorded only in 1 case of recurrent PA in the submandibular gland after intravenous contrast injection, which showed inhomogeneous enhancement [7]. The pattern of enhancement in our reported case was a partial inhomogeneous enhancement, as reported in previous reports. This pattern supported our pathologic findings. The pathologic findings showed fibrosis and necrosis in some of the lesions. This was also consistent with the previously reported pathologic findings of recurrent PA multi-nodularity in the parotid gland, i.e., the enhancement patterns showed a variety of findings ranging from solid, homogeneous, and heterogeneous to marginal enhancement (cystic pattern) [11].

Nodular lesions in the submandibular gland region are increasingly being detected in radiologic practice due to the increasing number of routine images. Recurrent PA is a type of nodule, but diagnostic imaging is challenging due to its rarity and lack of definitive imaging features. The differential diagnosis of recurrent PA includes lymphoepithelial carcinoma of the submandibular gland [12], adenoid cystic carcinoma [13], lymph node tuberculosis [14], and lymphoma [15]. The diagnosis of lymphoepithelial carcinoma of the submandibular gland and adenoid cystic carcinoma is prioritized if most of the nodal borders are indistinct. If most of the nodules show uneven peripheral enhancement and there is a history of tuberculosis, lymph node tuberculosis may be a priority. If multiple nodules in the submandibular gland region exhibit uniform density or signal and are associated with a history of Sjögren's syndrome, lymphoma may be prioritized [15].

Radiotherapy for the recurrence of pleomorphic adenomas has become a topic of consideration for scholars. Witt *et al*. summarized local tumor control after postoperative radiotherapy. Radiotherapy seems to improve the treatment outcomes of patients with multinodular tumors with multiple recurrences [16]. However, conclusions about the indications for radiotherapy are difficult to summarize. The side effects of radiotherapy and whether radiotherapy will induce the growth of other nodules in the future should also be considered. K. Aro *et al*. speculated that more extensive utilization of postoperative radiotherapy may delay further recurrences and suggested that postoperative radiotherapy appears to be necessary for patients with multifocal recurrent disease [17]. However, radiotherapy was not performed in our case.

## CONCLUSION

In conclusion, if the lesions in the submandibular gland area are multinodular and the pattern of enhancement varies from one nodule to another, recurrent PA should be included in the differential diagnosis. It is advisable to conduct a thorough history and physical examination to ascertain the patient's medical history and to elicit any pertinent information that may assist in making an accurate diagnosis.

## AUTHORS’ CONTRIBUTION

The authors confirm their contribution to the paper as follows: study conception and design: ZQL, YCX, and XWZ; data collection: GYY, YZ, and JBH; analysis and interpretation of results: ZQL, GYY, and FX; draft manuscript: ZQL.

All authors reviewed the results and approved the final version of the manuscript.

## Data Availability

The data and supportive information are available within the article.

## LIST OF ABBREVIATIONS

PA: Pleomorphic Adenoma
MRI: Magnetic Resonance Imaging
ADC: Apparent Diffusion Coefficient
CT: Computed Tomography
DWI: Diffusion-Weighted Imaging

## STANDARDS OF REPORTING

CARE guidelines were followed.
